# Social engagement in dementia: Activities, motivation, support, barriers, and increasing aspects

**DOI:** 10.1177/13872877251411341

**Published:** 2026-01-22

**Authors:** Hanna L. Knecht, Francisca S. Rodriguez

**Affiliations:** 1 German Center for Neurodegenerative Diseases (DZNE), RG Psychosocial Epidemiology & Public Health, Greifswald, Germany

**Keywords:** Alzheimer's disease, dementia, non-pharmacological interventions, psychosocial interventions

## Abstract

**Background:**

Social engagement has been connected to better psychological well-being, improved QoL, and resilience to neuropathological changes. Yet, little is known about the details of social engagement in dementia, which could inform effective interventions.

**Objective:**

With this study, we aimed at providing information on social engagement of people with dementia (PWD), given by proxies and PWD.

**Methods:**

501 people actively involved in dementia care in Germany (86% female; mean age 53.5 years) provided answers to a structured, quantitative survey via online/paper questionnaire, or interview on (i) the types of social activities that PWD engage in, (ii) PWDs’ motivation for social engagement, (iii) the support PWD get to engage, (iv) barriers to engage in activities, and (v) ways to increase social engagement. Descriptive analyses as well as overall and pairwise comparisons were performed.

**Results:**

PWD often attend therapies (M = 3.6, SD = 1.0) and sometimes meet-ups with friends (M = 3.0, SD = 1.0), and they remain interested in social engagement (M = 3.5, SD = 1.7). Support was perceived to come mainly from family members (88.9%), partners/spouses (85.9%), and friends/acquaintances (59.9%). Most participants perceived activities not being dementia-friendly (16.1%) and the lack of support (25.6%) as a major barrier to social engagement. To increase the engagement of PWD, participants suggested that social activities need to be adapted to their abilities (83.1%), that the community needs to provide inclusive activities (75.0%), and that specialized care services need to be expanded (41.4%).

**Conclusions:**

To facilitate and increase social engagement of PWD, support from social contacts and inclusive community behavior could be valuable steps.

## Introduction

The worldwide aging of societies is accompanied by an increase in dementia cases.^1^ Dementia is characterized by memory deficits, difficulties in speech and communication, and neuropsychiatric symptoms,^2^ as well as a decrease in the ability to manage everyday life independently, which eventually leaves PWD (people with dementia) depending on others’ support.^3^ Since the numbers of dementia cases will greatly rise,^4^ all involved in dementia care will face a great burden put on them, and successful interventions are needed urgently.^5^

Recent research emphasizes the importance of psychosocial factors in prevention and intervention.^6^ Especially social engagement, which entails participating in social activities and maintaining social connections,^7^ has been discussed in the wider literature as having a promising positive impact on several aspects of dementia. The state of research shows that a low level of social engagement, assessed through the frequency of social activity participation and social contact, is associated with an increased risk of developing dementia^8^ and with incident dementia.^9^

Moreover, being socially engaged can improve the PWDs’ psychological well-being. In fact, one research project found that PWD who had low levels of participation in social activities had more behavioral and psychological symptoms of dementia, which worsened over the course of a year, especially in those who had only little communication with family members.^10^ There has also been evidence that social isolation in PWD is associated with showing agitation^11^ and that overall social participation among nursing home residents with cognitive impairment is associated with a good quality of life.^12^

While benefits for people of old age and PWD of being socially engaged are documented, little is known about the aspects of current social engagement among PWD. That knowledge would be valuable for designing interventions that could positively affect the life of PWD. Therefore, the aim of this study was to provide information on (i) activities that PWD engage in, (ii) PWDs’ motivation for social engagement, (iii) the support PWD get to engage, (iv) barriers to engage in activities, and (v) ways to increase social engagement. To obtain an overview of the current state of affairs regarding the above points, we asked people who are involved in dementia care (e.g., family and professional caregivers, care coordinators) as well as PWD about their overall impression.

## Methods

The Ethics Committee of the University Medical Center Greifswald (No. B017/23) approved the study and written informed consent was obtained from the participants. To describe the current social situation of PWD living in Germany, people involved in dementia care, including spouses, partners in-/outside the home of the person with dementia home, caregivers, coordinators and staff in local alliances for PWD, volunteers, full-time employees at facilities for PWD, and PWD themselves were invited to participate in our structured quantitative survey.

### Sample

Recruitment was carried out via mail, flyer, newsletter, and social media. Throughout Germany, we forwarded the study information to dementia networks, self-help and interest groups, professional associations, dementia advocates, and other institutions connected to dementia care. To reach socially disadvantaged people and those with a migration background, connection was established to networks, institutions, and associations that represent those groups. Inclusion criteria were (i) 18 years of age and older, (ii) ability to give consent, which excludes people in a delirium or with an impaired consciousness, (iii) sufficient vision, and (iv) being actively involved in dementia care. Informed consent to participate was obtained from the participants. Information on the sociodemographic characteristics of the participants, which were on average 53.5 years of age (SD = 12.4, range 19–91), is presented in Table 1. Of the participants who self-reported a dementia diagnosis, two indicated vascular dementia and three reported Alzheimer's disease. Furthermore, five participants reported being in an early stage, while one reported being in a more advanced stage.

**Table 1.** Sociodemographic characteristics of the participants; n = 501.

**Characteristic**	**n**	**%**
Gender:		
Female	418	85.8
Male	69	14.2
Highest educational level:		
High school diploma or higher education	303	64.7
Middle school or lower education	165	35.3
Living environment:		
Urban	259	53.1
Rural	229	46.9
Income:		
High	48	9.9
Middle	398	82.4
Low	37	7.7
Cultural identity other than German:		
Yes	49	10.0
No	439	90.0
Other cultural identity:		
European	23	50.0
Asian	8	17.4
Middle Eastern	7	15.2
Role in dementia care:		
Family caregiver	182	38.3
Caring for someone outside their own home	104	21.9
Caring for someone at home	78	16.4
Professional caregiver	145	30.5
** **Others	135	28.4
Employee in counselling people with dementia	99	20.8
Therapist	25	5.3
In other ways involved in dementia care	11	2.3
** **Person with dementia	14	2.9

n: number of participants.

### Data collection

Throughout the period from August 15, 2023 to February 15, 2024, participants could partake in the study by choosing one of the following formats containing the same set of questions: online survey (95.6% of participants), paper questionnaire (3.2% of participants), and home or telephone interview (1.2% of participants).

### The questionnaire

The aim was to obtain a comprehensive overview of social engagement of PWD as perceived by various stakeholders. To obtain a broad perspective, a structured questionnaire was generated by researchers of this research group based on extracted information from the scientific literature and previously published surveys on this topic. Questions matching the study's aims were gathered, the most appropriate response mode for each question was chosen and their suitability for inclusion in the questionnaire was discussed. As PWD reported about their own experiences, one version of the questionnaire was specifically adapted to them. The general version of the questionnaire and the one for PWD were revised both internally and by five external experts for completeness, comprehensibility, and length. Changes based on the revisions included extending the list of options for single and multiple choice questions, adding examples, or merging similar questions to shorten the questionnaire.

The first questions of the questionnaire inquired about personal information such as age, gender, education, income, role in dementia care, urban/rural location, and cultural identity. PWD were encouraged to specify their type and stage of dementia in a free text field.

To identify activities that PWD engage in, all participants were subsequently asked how often [Likert scale: never (1) to always (5)] PWD can still participate at (i) meet-ups with friends, (ii) sporting activities, (iii) activities from associations, (iv) communal activities, (v) religious activities, (vi) support groups, (vii) cultural activities, and (viii) therapies.

To describe PWDs’ motivation for social engagement, solely PWD were further asked whether they agreed [Likert scale: strongly disagree (1) to strongly agree (5)] that (i) they have always been comfortable spending time on their own and only having a few social contacts, (ii) it has always been important for them and their general well-being to interact with others, (iii) since having dementia, it strains them to be among people, (iv) despite having dementia, they are interested in social interaction, (v) since having dementia, they prefer being alone, and (vi) they feel like undertaking something with others.

To assess barriers, participants were asked (i) in a single choice question what the most important barrier is that keeps PWD from engaging in out-of-home social activities, and (ii) whether the fear of making mistakes due to memory difficulties influences PWDs’ motivation to take part in social activities [Likert scale: not at all (1) to very much (5)].

To depict the support PWD get to engage in activities, we posed multiple choice questions on (i) which social contacts support PWD in organizing and undertaking social activities (e.g., hobbies) and (ii) what is considered necessary for PWD to still be able to engage in such social activities.

To assess what aspects may help to increase social engagement of PWD, we asked three types of questions. First, participants were invited to describe in a free text field (i) what has to change for PWD to maintain social relationships and to participate in communal social activities, and (ii) which social activities are lacking. Second, participants were asked how important [Likert scale: not at all (1) to very (5)] it is for the course of the disease that social activities are prescribed and arranged in the medical setting. Third, participants (only those without dementia) were asked whether they themselves have created social experiences for PWD before and if so, what kind of experiences [yes, no, free text].

### Statistical analysis

We conducted statistical analyses using STATA version 17.

In preparation for calculations, two researchers individually assigned categories to the information provided in free text fields and in the answers given to the option “other” of multiple choice questions. After having reached consensus on discrepancies, corresponding frequencies were calculated. Subsequently, we calculated frequencies and mean values for all questions. Further analyses were carried out to identify significant differences between (i) PWD, (ii) family caregivers (i.e., people caring for another at home and people caring for another outside their own home), (iii) professional caregivers, and (iv) people with other roles in dementia care (e.g., employee at a counselling service and therapists). Similarly, we identified significant differences between participants with a cultural identity other than German and those without. Results of the overall comparisons were displayed if cell count was n ≥ 5. Suppression due to low cell count was further applied in the pairwise comparisons.

## Results

Our findings are presented under the five aims of the study (engagement in social activities, PWDs’ motivation for social engagement, barriers and support to engage in activities, ways to increase social engagement), followed by differences between participant groups.

### Engagement in social activities

**Figure 1.**
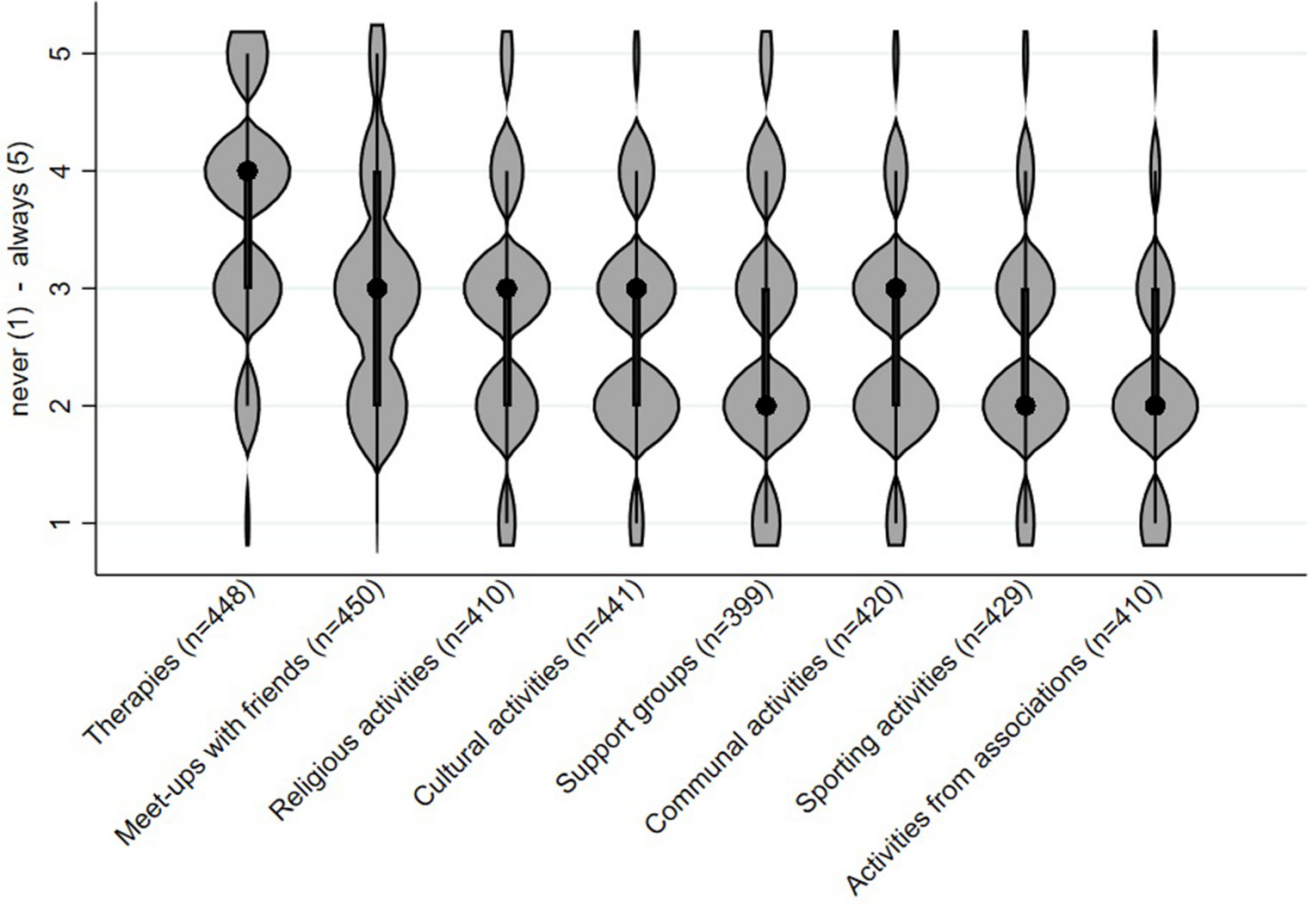
Likert scale answer from never (1) to always (5) on how often people with dementia participate in certain social activities. Black dots represent medians; thick black bars indicate interquartile ranges; violin width reflects data density.

Findings on the frequencies of the social activities that PWD still engage in are depicted in Figure 1. As our participants, mainly caregivers, indicated, PWD *often* take part in therapies (M = 3.6, SD = 1.0) and *sometimes* in meet-ups with friends (M = 3.0, SD = 1.0). Other social activities are carried out less frequently (see Figure 1).

### People with dementias’ motivation for social engagement

**Figure 2.**
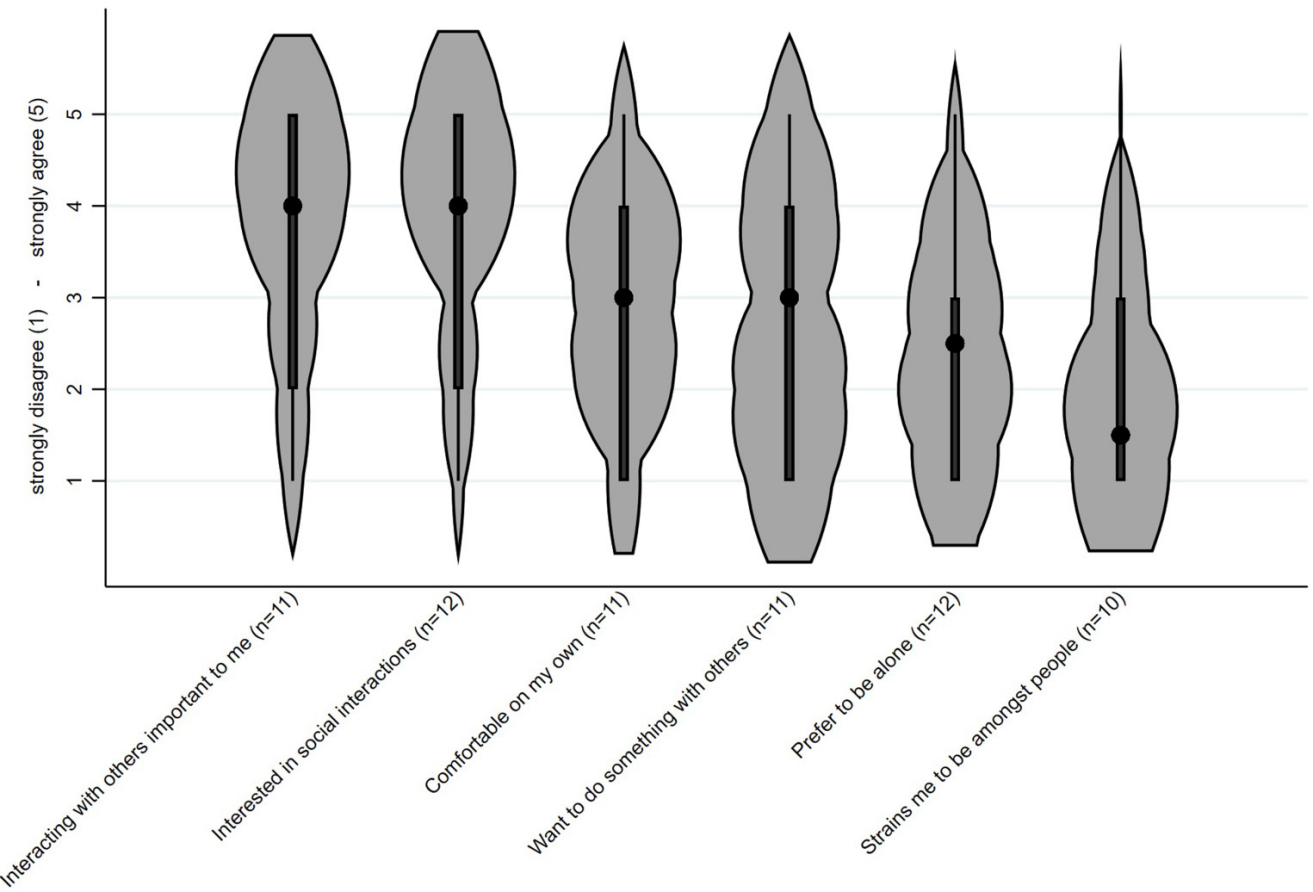
Likert scale answers from strongly disagree (1) to strongly agree (5) on whether participants with dementia agree with the statements about their motivation for social engagement. Black dots represent medians; thick black bars indicate interquartile ranges; violin width reflects data density.

The answers of the participants with dementia concerning their own motivation to engage socially are shown in Figure 2. They *tended to disagree* that they prefer being alone (M = 2.4, SD = 1.3) and that, since having dementia, it strains them to be amongst other people (M = 2.1, SD = 1.2; see Figure 2). Moreover, they *tended to agree* that it has always been important for them and their general well-being to interact with others (M = 3.6, SD = 1.6) and that, despite having dementia, they are interested in social interactions (M = 3.5, SD = 1.7; see Figure 2).

### Barriers to engage in activities

The plurality of participants, of which most were caregivers, chose the lack of support (25.6%) as the most important barrier for PWD to engage in out-of-home activities, followed by activities not being dementia-friendly (16.1%) and the fear of losing orientation (15.3%; see Table 2). Participants further indicated that the fear of making mistakes due to memory difficulties *substantially* influences (M = 4.1; SD = 1.0) PWDs’ motivation of engaging in social activities.

**Table 2.** Participants’ answers to questions about different aspects of social engagement; n = 501.

Question	Answer	% (n/N)
Barriers to engage in activities	Lack of support	25.6 (94/367)
	Activities not being dementia-friendly	16.1 (59/367)
	Fear of losing orientation/ lack of feeling safe	15.3 (56/367)
	Fear of revealing dementia	11.4 (42/367)
	Great organizational effort	9.3 (34/367)
	Lack of PWDs’ motivation	7.9 (29/367)
	Lack of means of transportation	4.6 (17/367)
	Limited physical strength	3.3 (12/367)
	Fear of rejection	2.5 (9/367)
	Financial situation	2.2 (8/367)
Social contacts that support PWD to engage in activities	Family member	88.9 (352/396)
	Partner or spouse	85.9 (340/396)
	Friend and acquaintance	59.9 (237/396)
	Professional caregiver	49.8 (197/396)
	Volunteer	46.0 (182/396)
	Acquaintance from activities/ counselling for PWD	43.7 (173/396)
	Neighbor	31.3 (124/396)
	Therapist	26.3 (104/396)
	Support group participant	21.0 (83/396)
	Doctor	12.9 (51/396)
	Service provider (e.g., vendor)	3.3 (13/396)
	Social contact from work or school	2.8 (11/396)
	User of social media (e.g., Facebook)	2.8 (11/396)
	Care services staff organizing social activities	1.8 (7/396)
Requirements to engage in activities	Adapting activities to the abilities of PWD	83.1 (329/396)
	Inclusive behavior of the community	75.0 (297/396)
	Support in transportation	73.5 (291/396)
	PWDs’ motivation	71.0 (281/396)
	Support conducting the activity	70.7 (280/396)
	Support getting dressed	47.0 (186/396)
	Supportive basic conditions (e.g., financially viable)	3.8 (15/396)
Needed changes^a^	Offering inclusive activities	46.3 (124/268)
	Expanding various care services (e.g., 1:1 assistance)	41.4 (111/268)
	Improving basic conditions (e.g., time resources)	15.7 (42/268)
	Financial relief (e.g., increase care reimbursement)	9.0 (24/268)
	Recognizing and strengthening voluntary work	6.0 (16/268)
	Extending activities for young PWD	4.5 (12/268)
	Raising awareness in public institutions	4.5 (12/268)
	Improving means of transportation	4.5 (12/268)
	Extending activities in rural areas	3.4 (9/268)
	Creating rooms for encounters	3.0 (8/268)
Lacking social activities	Social activities specifically for PWD	72.7 (165/227)
	Inclusive activities (e.g., several generations joining)	13.2 (30/227)
	More flexible care services	12.3 (28/227)
	Day care or short-term care services	7.5 (17/227)
	All	5.3 (12/227)
	Support groups	4.9 (11/227)
	Activities with flexible means of transportation	4.9 (11/227)
	Activities for young PWD	4.0 (9/227)
	Financially viable activities	3.5 (8/227)
Social experiences created for PWD^$^	Organizing leisure activities	39.7 (92/232)
	Creating support groups or alike	22.0 (51/232)
	Inviting to social activities in private settings	21.6 (50/232)
	Organizing activities adapted to personal interests	14.7 (34/232)
	Enabling participation in public events (e.g., concerts)	12.1 (28/232)
	Organizing a range of different therapies	7.8 (18/232)
	Organizing vacation and trips	5.6 (13/232)
	Creating animal-assisted activities	3.0 (7/232)

^a^ for maintaining social relationships & participating in activities of the community; n: number of participants choosing the particular answer; N: number of participants answering the question; PWD: people with dementia; ^$^question solely for participants without dementia.

### Support to engage in activities

The majority of respondents, of which most were caregivers, indicated that family members (88.9%), partners or spouses (85.9%), and friends or acquaintances (59.9%) are most often the social contacts that support PWD in organizing and undertaking social activities (see Table 2). Furthermore, for being able to engage in social activities, most participants considered it necessary to adapt activities to the cognitive and physical abilities of PWD (83.1%), that there is an inclusive behavior in the community (75.0%), to have transportation (73.5%), that PWD are motivated (71.0%), and that PWD are supported in conducting the activity (70.7%; see Table 2).

### Increasing social engagement

#### Needed changes

The frequencies of the responses indicate that most participants perceive that, for PWD to maintain social relationships and to participate in activities of the community, the following changes are needed: offering inclusive activities (46.3%), expanding various care services such as one on one assistance (41.4%), and improving conditions of qualified personnel such as more time (15.7%; see Table 2). When asked about what social activities are lacking, the majority of participants selected social activities specifically for PWD (72.7%), inclusive activities such as activities bringing together several generations (13.2%), and more flexible care services (12.3%; see Table 2). Furthermore, participants responded on average that it is *important* (M = 4.0, SD = 1.1) for the course of the disease that engagement in social activities is initiated in the medical setting.

#### Creation of social activities

Nearly three quarters of participants without dementia (73.5%) confirmed that they have created social experiences for PWD in the past. As shown in Table 2, most of them were organized leisure activities (39.7%). More than a fifth of the participants furthermore mentioned that they created support groups or alike (22.0%) and invited PWD to social activities in private settings such as birthday parties (21.6%).

### Differences between participant groups

**Figure 3.**
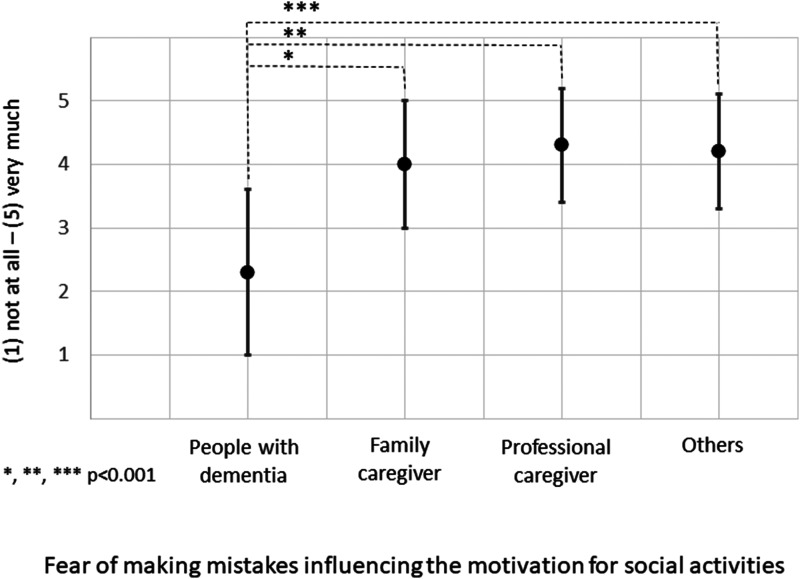
Differences in answers between people with dementia and other participant groups on the influence of fear of making mistakes on the motivation for social activities; statistically significant differences from pairwise comparisons. Black dots indicate mean values of Likert scale answers from (1) not at all to (5) very much; black bars indicate standard deviations.

Details of the pairwise comparison by participant group according to their role in dementia care are shown in Supplemental Tables 1 and 2. Merely a few statistically significant results were detected. Statistically significant differences indicated that professional caregivers, and to a smaller extent those otherwise involved in dementia care, described a higher frequency of engagement in several activities (e.g., sports and community activities). They furthermore reported more often than family caregivers that PWD receive support from acquaintances from activities/ counselling for PWD and that they created leisure activities. In comparison to other participant groups, PWD perceived less support through family caregivers, a smaller importance of arranging social engagement in the medical setting, a smaller necessity for adapting social activities specifically for them, and a smaller influence of the fear of making mistakes on the motivation to participate socially (see Figure 3).

Results from the comparison of participants with German cultural identity and those with other cultural identities are shown in the Supplemental Tables 4 and 5. Only a few statistically significant results were identified. They show that more participants with another cultural identity than those with a German cultural identity chose more frequently doctors, support group participants, users of social media (e.g., Facebook), and service providers (e.g., vendor) as social contacts that support PWD to engage in social activities.

## Discussion

With this study, we aimed at obtaining an overview of (i) social activities that PWD engage in, (ii) PWDs’ motivation for socially engaging, (iii) support PWD get to participate socially, (iv) barriers to engage in social activities, and (v) aspects that can increase social engagement. Most of the respondents of the survey were caregivers (69%). Overall, the findings suggest that therapies and meetings with friends make up a major part of social activities that PWD still engage in, motivated by their continued interest in social participation and supported by family members, partners, and friends/acquaintances. To facilitate and to increase PWDs’ social engagement, participants indicated that an inclusive behavior in the community, provision of support (e.g., flexible care services, transportation), and offering activities that meet the needs and abilities of PWD are needed. According to the participants, those are, however, noticeably limited. Advocacy for arranging social engagement in the medical setting emphasizes that those actively involved in dementia care think that it is important that their attending physician should make local and suitable offers available to those living with dementia.

Caring for someone with dementia is often associated with an increased mental and physical burden on the primary caregiver,^13^ which can be reduced by support from other social contacts of the PWD. Interestingly, our findings show that family caregivers perceive less support from other social contacts than do professional caregivers. Possible explanations found in the wider literature include that family caregivers might feel hesitant to ask for or even reject support of others because they want to avoid being a burden to them, wish to remain independent, or want the bond with the family member with dementia to remain unchanged as long as possible.^14^ Other reasons for perceiving less support may include family caregivers’ self-expectations related to fulfilling a new identity as caregiver keeping them from asking for support,^15^ as well as feelings of stigmatization in the society.^16^ Some family caregivers might have even experienced withdrawal of close and important people after the dementia diagnosis, which made them feel insecure about the support they would receive and made them protective of themselves and their family member.^17^ On the other hand, neighbors, acquaintances from activities for PWD, support-group participants, and others that could provide support may refrain from it because they do not want their relationship with the family caregiver or the person with dementia to change, or because they fear of unintentionally behaving in a stigmatizing way.^14^ Unlike family caregivers, professional caregivers may not face the aforementioned barriers, as they are mainly the result of having previously had a personal and/or emotional relationship with the person with dementia. In addition, professional caregivers may be more aware of social support resources, whereas there seems to be a lack of knowledge among family caregivers about social support services that could be contacted, as suggested in a research article about caregivers’ knowledge about social support.^18^

Consistent with the wider literature, our findings indicate that an inclusive behavior in the community is essential for PWD to engage in social activities. It is suggested that PWD discontinue their engagement due to fear of judgement^2^ or due to getting the feeling of not belonging,^19^ which might be more common in communities that are not particularly dementia friendly. PWD feel motivated to participate in a social setting when their contribution to and participation in the community is perceived meaningful and valued.^19^ Additionally, a community acting in an inclusive manner enables and facilitates the engagement of PWD by empowering and involving them in creating social activities in the community they feel safe with,^20^ where they are supported,^20^ and in which they themselves can participate.^21^ Inclusive communities might also be more aware of and proactively adapt activities to cognitive and physical abilities of PWD,^22^ be more invested in promoting networks of support that meet PWDs’ specific needs,^20^ and reduce the burden that is mostly put on family members and spouses.^23^ In conclusion, it is essential to foster public awareness of and knowledge about dementia in order to enable engagement of PWD.^19^

Due to a number of barriers, PWDs’ need for social engagement is not or insufficiently met.^24^ According to our findings and the wider literature, the lack of support poses a significant hindrance for PWD to access and participate in social out-of-home activities. As dementia progresses, this becomes increasingly difficult, until the obstacles cannot be tackled without support from social contacts.^25^ This can, for instance, be instrumental support such as driving PWD to activities or support on an emotional basis such as PWD being motivated by others to participate (e.g., encourage PWD to leave the house).^26^ A common conclusion in research is that PWD want to and can engage in social activities if sufficient social support is provided by family caregivers, friends, and the wider community,^2^ but that there often is a discrepancy between the needs and the availability of support^20^ due to, amongst other things, limitations in financial means.^27^ Also, activities need to match the PWDs' abilities, which may not be the case if the activities go beyond those planned and executed with the care partners.^28^ Engaging in social activities is usually further restricted by challenges such as orientating oneself, engaging with unfamiliar people, and not feeling safe^25^ or rather the fear of making a mistake.^29^ Ultimately, the challenges must be overcome by PWD who have the support of social contacts and must be minimized by the community as a whole.

Limitations of this study include that, despite our best efforts, the subjective experiences of PWD are greatly under-represented (3%) and that, although participants could choose between three different survey formats aimed at including a variety of people, selection bias must be considered. The predominant use of the online survey format may have limited the depth of responses and may have introduced the possibility of misunderstandings that an interviewer could have clarified. Selection bias was further introduced by participant recruitment via networks, which may have excluded less connected PWD. Their responses could have offered valuable insights into the experiences of individuals with presumably lower levels of social engagement in organized settings. Moreover, the use of subjective, retrospective methods such as self-report might not concur with objective measurements such as trackers, diaries, or journals. The findings may be biased in that respect. We emphasize that the findings are to be interpreted as subjective experiences of PWD and mainly of others active in dementia care but are nonetheless important to highlight what has to be done to improve the situation of PWD. Furthermore, we want to highlight the necessity of investigating the differences regarding the stage and type of dementia in future studies. It further should be acknowledged that the questionnaire, which we believe provided valuable information, is not a validated tool. Consequences may include limitations in making comparisons with other studies. However, the aim was to gain a comprehensive overview of the situation of PWD and not just measure one construct within one specific group. Importantly, experts reviewed the questionnaire prior to data collection. Biases that may have been introduced by presenting participants with a selection of responses were minimized by always giving the opportunity to add own thoughts in write-in options. Further strengths of our study include its large and diverse sample size that enhances generalizability and the use of free text questions, which allows deeper insights into social health issues. Middle-aged women predominantly engage in caregiving and work in care settings, making their predominance among our participants a strength.^30^ In addition, subjective measures that rely on self- and proxy reports offer the opportunity to gather lived experiences, which are important in dementia care. Future research should acknowledge both limitations and strengths.

### Conclusion

This study provides information on the barriers and the supportive contacts of PWDs’ engagement and on how participation might be increased. Our findings emphasize the eagerness of most PWD to continue engaging in social activities, but also highlight the challenges such as lack of support and limited provision of tangible activities. There is a need for an inclusive behavior by the community, which means enabling PWD to participate in diverse activities and offering a variety of care services. Finally, we would like to point out the necessity of further research to explore methods to proactively provide opportunities for social engagement of PWD.

## Supplemental Material

sj-docx-1-alz-10.1177_13872877251411341 - Supplemental material for Social engagement in dementia: Activities, motivation, support, barriers, and increasing aspects Supplemental material, sj-docx-1-alz-10.1177_13872877251411341 for Social engagement in dementia: Activities, motivation, support, barriers, and increasing aspects by Hanna L. Knecht and Francisca S. Rodriguez in Journal of Alzheimer's Disease
