# Supplementary material for: Social engagement in dementia: Activities, motivation, support, barriers, and increasing aspects
Source: J Alzheimers Dis. 2026 Jan 22;109(4):1874–83. doi: 10.1177/13872877251411341 (PMC12894425; doi:10.1177/13872877251411341)
Supplement: sj-docx-1-alz-10.1177_13872877251411341 - Supplemental material for Social engagement in dementia: Activities, motivation, support, barriers, and increasing aspects [file sj-docx-1-alz-10.1177_13872877251411341.docx]

**Supplemental Material**

**Social engagement in dementia: Activities, motivation, support, barriers, and increasing aspects**

**Supplemental Table 1.** Differences in Likert scale answers between people with dementia (PWD), family caregiver (FC), professional caregivers (PC), and people otherwise connected to dementia care (others); n=476

| **Question** | **Overall comparison** | | | | | | | | | | **Pairwise comparison*** | | | | | | |  |
| --- | --- | --- | --- | --- | --- | --- | --- | --- | --- | --- | --- | --- | --- | --- | --- | --- | --- | --- |
|  | PWD (n=14) | | FC (n=182) | | | PC (n=145) | | Others (n=135) | |  | | FC - PWD | PC - PWD | Others - PWD | FC - PC | FC - Others | PC - Others | |
|  | n | M±SD | n | M±SD | n | | M±SD | n | M±SD | X²(p) | | M∆, p | M∆, p | M∆, p | M∆, p | M∆, p | M∆, p | |
| **Frequency of participation** |  |  |  |  |  | |  |  |  |  | |  |  |  |  |  |  | |
| Meet-ups with friends | 12 | 3.1±1.1 | 169 | 3.0±1.0 | 134 | | 3.2±1.1 | 123 | 2.9±0.8 | 5.472 (0.143) | |  |  |  |  |  |  | |
| Sporting activities | 11 | 2.6±1.5 | 154 | 2.4±1.0 | 131 | | 2.7±1.0 | 122 | 2.5±0.7 | 9.726 (0.021) | | 0.2, 0.055 | 0.2, 0.538 | 0.1, 0.746 | 0.4, 0.001 | 0.1, 0.474 | 0.3, 0.019 | |
| Activities from associations | 11 | 1.8±1.4 | 142 | 2.1±0.9 | 127 | | 2.4±1.0 | 119 | 2.4±0.7 | 17.208 (<0.001) | | 0.3, 0.388 | 0.6, 0.044 | 0.6, 0.047 | 0.3, 0.003 | 0.3, 0.003 | 0.0, 0.951 | |
| Communal activities | 11 | 1.8±1.3 | 146 | 2.4±0.9 | 129 | | 2.8±1.0 | 123 | 2.7±0.7 | 16.429 (<0.001) | | 0.6, 0.027 | 1.0, 0.001 | 0.9, 0.003 | 0.4, 0.001 | 0.2, 0.031 | -0.1, 0.267 | |
| Religious activities | 10 | 1.5±1.3 | 142 | 2.7±1.1 | 131 | | 3.1±1.0 | 117 | 2.8±0.8 | 27.635 (<0.001) | | 1.2, <0.001 | 1.6, <0.001 | 1.3, <0.001 | 0.5, <0.001 | 0.1, 0.310 | 0.3, 0.005 | |
| Support groups | 12 | 2.7±1.7 | 142 | 2.5±1.2 | 118 | | 2.8±1.1 | 117 | 2.7±0.9 | 3.201 (0.362) | |  |  |  |  |  |  | |
| Cultural activities | 13 | 2.8±1.5 | 163 | 2.7±1.0 | 131 | | 2.9±1.0 | 123 | 2.6±0.8 | 4.706 (0.195) | |  |  |  |  |  |  | |
| Therapies | 11 | 2.8±1.4 | 167 | 3.5±1.1 | 134 | | 3.8±0.9 | 124 | 3.6±0.8 | 8.743 (0.033) | | 0.7, 0.031 | 1.0, 0.002 | 0.8, 0.016 | 0.3, 0.009 | 0.1, 0.463 | -0.2, 0.085 | |
|  |  |  |  |  |  | |  |  |  |  | |  |  |  |  |  |  | |
| **Fear of making mistakes influences motivation for social activities** | 12 | 2.3±1.3 | 148 | 4.0±1.0 | 110 | | 4.3±0.9 | 99 | 4.2±0.9 | 23.106 (<0.001) | | 1.7, <0.001 | 2.0, <0.001 | 1.9, <0.001 | 0.3, 0.017 | 0.2, 0.114 | 0.1, 0.481 | |
|  |  |  |  |  |  | |  |  |  |  | |  |  |  |  |  |  | |
| **Need to arrange social engagement in medical setting** | 11 | 2.7±1.7 | 151 | 3.9±1.2 | 111 | | 4.1±1.1 | 105 | 4.2±0.9 | 8.830 (0.032) | | 1.2, 0.001 | 1.3, <0.001 | 1.4, <0.001 | 0.2, 0.239 | 0.3, 0.037 | 0.1, 0.385 | |

*only if overall comparison was p<0.05; FC: family caregiver; M: mean; M∆, difference in mean; n: number of participants; p: level of significance; PC: professional caregiver; PWD: people with dementia; SD: standard deviation; x2: chi square as of Kruskall-Wallis test.

**Supplemental Table 2.** Differences in frequencies of answers between people with dementia (PWD), family caregiver (FC), professional caregivers (PC), and people otherwise connected to dementia care (others); n= 476

| **Question** | **Answer** | **Overall comparison** | | | | | | **Pairwise comparison *^,§^** | | | | | |
| --- | --- | --- | --- | --- | --- | --- | --- | --- | --- | --- | --- | --- | --- |
|  |  | PWD (n=14) | FC (n=182) | PC (n=145) | Others (n=135) |  | FC - PWD | | PC - PWD | Others - PWD | FC - PC | FC - Others | PC - Others |
|  |  | % (n) | % (n) | % (n) | % (n) | X² (p) | X² (p) | | X² (p) | X² (p) | X² (p) | X² (p) | X² (p) |
| **Social contacts that support PWD to engage in activities** |  |  |  |  |  |  |  | |  |  |  |  |  |
| Family member | Yes  No | 41.7 (5) 58.3 (7) | 91.5 (140) 8.5 (13) | 92.1 (105) 7.9 (9) | 92.1 (93) 7.9 (8) | 33.332 (<0.001) | 25.945 (<0.001) | | 24.916 (<0.001) | 23.678 (<0.001) | 0.031 (0.860) | 0.027 (0.870) | 0.000 (0.994) |
| Friend and acquaintance | Yes  No | 50.0 (6) 50.0 (6) | 59.5 (91) 40.5 (62) | 63.2 (72) 36.8 (42) | 62.4 (63) 37.6 (38) | 1.063 (0.786) |  | |  |  |  |  |  |
| Acquaintance from activities/  counselling for PWD | Yes  No | 41.7 (5) 58.3 (7) | 30.7 (47) 69.3 (106) | 48.3 (55) 51.8 (59) | 60.4 (61) 39.6 (40) | 22.803 (<0.001) | 0.618 (0.432) | | 0.188 (0.664) | 1.549 (0.213) | 8.500 (0.004) | 21.924 (<0.001) | 3.182 (0.074) |
|  |  |  |  |  |  |  |  | |  |  |  |  |  |
| **Requirements to engage in activities** |  |  |  |  |  |  |  | |  |  |  |  |  |
| Support in transportation | Yes  No | 50.0 (6) 50.0 (6) | 77.8 (119) 22.2 (34) | 71.9 (82) 28.1 (32) | 75.3 (76) 24.8 (25) | 5.079 (0.166) |  | |  |  |  |  |  |
| Inclusive behavior of the  community | Yes  No | 50.0 (6) 50.0 (6) | 68.0 (104) 32.0 (49) | 79.8 (91) 20.2 (23) | 86.1 (87) 13.9 (14) | 16.350 (0.001) | 1.618 (0.203) | | 5.451 (0.020) | 9.616 (0.002) | 4.658 (0.031) | 10.763 (0.001) | 1.499 (0.221) |
| Adapting activities to the  abilities of PWD | Yes  No | 50.0 (6) 50.0 (6) | 81.7 (125) 18.3 (28) | 86.8 (99) 13.2 (15) | 90.1 (91) 9.9 (10) | 14.696 (0.002) | 6.835 (0.009) | | 10.611 (0.001) | 14.189 (<0.001) | 1.279 (0.258) | 3.374 (0.066) | 0.553 (0.457) |
|  |  |  |  |  |  |  |  | |  |  |  |  |  |
| **Social experiences created for PWD^$^** | Yes  No |  | 66.9 (115) 33.1 (57) | 75.6 (102) 24.4 (33) | 78.6 (99) 21.4 (27) | 5.718 (0.057) |  | |  |  |  |  |  |
| Creating support groups  or alike | Yes  No |  | 14.6 (12) 85.4 (70) | 15.6 (10) 84.4 (54) | 34.2 (26) 65.8 (50) | 10.828 (0.004) |  | |  |  | 0.028 (0.868) | 8.275 (0.004) | 6.283 (0.012) |
| Enabling participation in  public events (e.g., concerts) | Yes  No |  | 12.2 (10) 87.8 (72) | 17.2 (11) 82.8 (53) | 7.9 (6) 92.1 (70) | 2.809 (0.246) |  | |  |  |  |  |  |
| Organizing activities adapted  to personal interests | Yes  No |  | 15.9 (13) 84.2 (69) | 18.8 (12) 81.3 (52) | 10.5 (8) 89.5 (68) | 1.957 (0.376) |  | |  |  |  |  |  |
| Organizing leisure activities | Yes  No |  | 25.6 (21) 74.4 (61) | 46.9 (30) 53.1 (34) | 50.0 (38) 50.0 (38) | 11.493 (0.003) |  | |  |  | 7.151 (0.008) | 10.028 (0.002) | 0.136 (0.712) |

*only if overall comparison was p<0.05; ^§^not reported if n<5; ^$^question solely for participants without dementia; FC: family caregiver; n, number of participants; p: level of significance; PC: professional caregiver; PWD: people with dementia; x2: chi square test.

**Supplemental Table 3.** Topics, questions, and the corresponding response formats

| Topic | Questions | Response method |
| --- | --- | --- |
| Engagement in social activities | “How often can PWD still participate at … ?”   - Meet-ups with friends - Sporting activities - Activities from associations - Communal activities - Religious activities - Support groups - Cultural activities - Therapies | Likert scale: never (1) to always (5), “not available” |
| People with dementias’ motivation for social engagement | “Do you agree … ?”^§^   - I have always been comfortable spending time on my own and only having a few social contacts - It has always been important for me and my general well-being to interact with others - Since having dementia, it strains me to be amongst people - Despite having dementia, I am interested in social interaction - Since having dementia, I prefer being alone - I feel like undertaking something with others | Likert scale: not very (1) to strongly (5) |
| Barriers to engage in activities | “What is the most important barrier that keeps PWD from engaging in out-of-home social activities?” | Single choice:   - Lack of support - Activities not being dementia-friendly - Fear of losing orientation/ lack of feeling safe - Fear of revealing dementia - Great organizational effort - Lack of PWDs’ motivation - Lack of means of transportation - Limited physical strength - Fear of rejection - Financial situation - Others: [free text] |
|  | “To what extent does the fear of making mistakes due to memory difficulties influence PWDs’ motivation to take part in social activities?” | Likert scale: not at all (1) to very (5) |
| Support to engage in activities | “Which social contacts support PWD in organizing and undertaking social activities (e.g., hobbies)?” | Multiple choice:   - Family member - Partner or spouse - Friend and acquaintance - Professional caregiver - Volunteer - Acquaintance from activities/ counselling for PWD - Neighbor - Therapist - Support group participant - Doctor - Service provider (e.g., vendor) - Social contact from work or school - User of social media (e.g., Facebook) - Others: [free text]   Additional choice for PWD: self-reflective self |
|  | “What is considered necessary for PWD to still being able to engage in such social activities?” | Multiple choice:   - Adapting activities to the abilities of PWD - Inclusive behavior of the community - Support in transportation - PWDs’ motivation - Support conducting the activity - Support getting dressed - Others: [free text] |
| Increasing social engagement | “What has to change for PWD to maintain social relationships and to participate in communal social activities?” | Free text |
|  | “Which social activities are lacking?” | Free text |
|  | “How important is it for the course of the disease that social activities are prescribed and arranged in the medical setting?” | Likert scale: not very (1) to very (5) |
|  | “Have you yourself created social experiences for PWD before and if so, what kind of experiences?”^$^ | Single choice:   - Yes - No   Free text |

^§^question solely for participants with dementia; ^$^question solely for participants without dementia.

**Supplemental Table 4.** Differences in Likert scale answers between participants with German cultural identity and participants with other cultural identities; n=488

| **Question** | With other cultural identities (n=49) | | With German cultural identity (n=439) | |  |
| --- | --- | --- | --- | --- | --- |
|  | n | M±SD | n | M±SD | z(p) |
| **Frequency of participation** |  |  |  |  |  |
| Meet-ups with friends | 44 | 3.0±1.0 | 404 | 3.0±1.0 | -0.272 (0.786) |
| Sporting activities | 42 | 2.6±0.9 | 386 | 2.5±0.9 | 0.393 (0.694) |
| Activities from associations | 42 | 2.4±0.9 | 366 | 2.2±0.9 | 1.393 (0.164) |
| Communal activities | 41 | 2.6±0.7 | 377 | 2.6±0.9 | 0.438 (0.662) |
| Religious activities | 41 | 2.9±1.0 | 367 | 2.8±1.0 | 0.455 (0.649) |
| Support groups | 42 | 2.8±1.2 | 356 | 2.6±1.1 | 0.517 (0.605) |
| Cultural activities | 44 | 2.8±0.9 | 395 | 2.7±0.9 | 0.445 (0.657) |
| Therapies | 45 | 3.5±0.9 | 401 | 3.6±1.0 | -0.292 (0.770) |
|  |  |  |  |  |  |
| **People with dementias’ motivation for social engagement^§^** |  |  |  |  |  |
| Despite having dementia, I am interested in social interaction | 1 | 5.0±0.0 | 11 | 3.4±1.7 | 1.063 (0.288) |
| Since having dementia, I prefer being alone | 1 | 1.0±0.0 | 11 | 2.6±1.3 | -1.204 (0.229) |
|  |  |  |  |  |  |
| **Fear of making mistakes influences motivation for social activities** | 36 | 4.2±1.0 | 340 | 4.1±1.0 | 0.312 (0.755) |
|  |  |  |  |  |  |
| **Need to arrange social engagement in medical setting** | 39 | 4.1±1.1 | 347 | 4.0±1.1 | 0.647 (0.517) |

^§^question solely for participants with dementia; n: number of participants; M: mean; SD: standard deviation; p: level of significance; z: z-score from Wilcoxon rank-sum test.

**Supplemental Table 5.** Differences in frequencies of answers between participants with German cultural identity and participants with other cultural identities; n=488

| **Question** | **Answer** | With other cultural identities (n=49) | With German cultural identity (n=439) |  |
| --- | --- | --- | --- | --- |
|  |  | % (n) | % (n) | X² (p) |
| **Social contacts that support PWD to engage in activities** |  |  |  |  |
| Partner or spouse | Yes  No | 86.1 (31)  13.9 (5) | 87.2 (307)  12.8 (45) | 0.036 (0.851) |
| Family member | Yes  No | 86.1 (31)  13.9 (5) | 90.6 (319)  9.4 (33) | 0.753 (0.385) |
| Friend and acquaintance | Yes  No | 69.4 (25)  30.6 (11) | 60.2 (212)  39.8 (140) | 1.167 (0.280) |
| Neighbor | Yes  No | 33.3 (12)  66.7 (24) | 31.8 (112)  68.2 (240) | 0.035 (0.853) |
| Professional caregiver | Yes  No | 61.1 (22)  38.9 (14) | 49.7 (175)  50.3 (177) | 1.697 (0.193) |
| Acquaintance from activities/counselling for PWD | Yes  No | 50.0 (18)  50.0 (18) | 43.8 (154)  56.3 (198) | 0.517 (0.472) |
| Doctor | Yes  No | 27.8 (10)  72.2 (26) | 11.7 (41)  88.4 (311) | 7.443 (0.006) |
| Therapist | Yes  No | 38.9 (14)  61.1 (22) | 25.6 (90)  74.4 (262) | 2.954 (0.086) |
| Support group participant | Yes  No | 41.7 (15)  58.3 (21) | 19.3 (68)  80.7 (284) | 9.701 (0.002) |
| User of social media (e.g., Facebook) | Yes  No | 13.9 (5)  86.1 (31) | 1.7 (6)  98.3 (346) | 17.601 (<0.001) |
| Service provider (e.g., vendor) | Yes  No | 13.9 (5)  86.1 (31) | 2.3 (8)  97.7 (344) | 13.609 (<0.001) |
| Volunteer | Yes  No | 52.8 (19)  47.2 (17) | 46.0 (162)  54.0 (190) | 0.599 (0.439) |
|  |  |  |  |  |
| **Requirements to engage in activities** |  |  |  |  |
| PWDs’ motivation | Yes  No | 75.0 (27)  25.0 (9) | 71.6 (252)  28.4 (100) | 0.188 (0.665) |
| Support getting dressed | Yes  No | 58.3 (21)  41.7 (15) | 46.3 (163)  53.7 (189) | 1.895 (0.169) |
| Support in transportation | Yes  No | 72.2 (26)  27.8 (10) | 74.7 (263)  25.3 (89) | 0.107 (0.744) |
| Support conducting the activity | Yes  No | 77.8 (28)  22.2 (8) | 71.0 (250)  29.0 (102) | 0.734 (0.392) |
| Inclusive behavior of the community | Yes  No | 86.1 (31)  13.9 (5) | 75.0 (264)  25.0 (88) | 2.213 (0.137) |
| Adapting activities to the abilities of PWD | Yes  No | 83.3 (30)  16.7 (6) | 84.4 (297)  15.6 (55) | 0.027 (0.870) |
|  |  |  |  |  |
| **Needed changes** |  |  |  |  |
| Expanding various care services (e.g., 1:1 assistance) | Yes  No | 40.0 (12)  60.0 (18) | 41.6 (99)  58.4 (139) | 0.028 (0.867) |
| Offering inclusive activities | Yes  No | 36.7 (11)  63.3 (19) | 47.5 (113)  52.5 (125) | 1.253 (0.263) |
|  |  |  |  |  |
| **Lacking social activities** |  |  |  |  |
| Social activities specifically for PWD | Yes  No | 65.0 (13)  35.0 (7) | 73.4 (152)  26.6 (55) | 0.653 (0.419) |
|  |  |  |  |  |
| **Social experiences created for PWD $** | Yes  No | 79.6 (35)  20.5 (9) | 72.8 (291)  27.3 (109) | 0.938 (0.333) |
| Organizing activities adapted to personal interests | Yes  No | 27.8 (5)  72.2 (13) | 13.7 (29)  86.3 (183) | 2.618 (0.106) |
| Organizing leisure activities | Yes  No | 50.0 (9)  50.0 (9) | 39.2 (83)  60.9 (129) | 0.814 (0.367) |

Not reported if n<5; ^$^question solely for participants without dementia; n: number of participants; p: level of significance; x2: chi square test.
